# Bone, Joint, and Orthopædic Surgery

**Published:** 1894-08-25

**Authors:** 


### PROGRESS IN SURGERY.

### BONE, JOINT, AND ORTHOPÆDIC SURGERY.

Under the term sub-articular necrosis C. T. Dent[1] describes a case of effusion into the knee joint arising from a tuberculous abscess in the head of the tibia, which was opened freely. At the time of writing there was every prospect of complete recovery in the joint. He remarks, "if left alone sequestra in such abscess cavities exhibit a marked tendency to travel, and especially in the upper end of the ulna. The travelling is effected by the agency of vascular granulations which spring up behind and push the loose piece of bone in the direction of the joint cavity." Dent adds a similar condition, as far as its results go, has been described by T. Smith under the term, Acute Arthritis of Infants. In the latter disease the progress is osteo-myelitic rather than tubercular. Griffiths and Milligan[2] describe a case of ossification of the synovial membrane of the left knee joint in a man aged twenty-three. The knee was injured at the age of fourteen years, and synovitis, apparently tubercular, supervened. This became quiescent, leaving the membrane thickened. The joint was resected, and bony plates were found in the synovial pouch above the knee with considerable lipping of the margins of the articular cartilage. The authors advance the opinion that it was a true case of ossification occurring in the connective tissue of the thickened synovial membrane.

**Bier's Method of Treating Strumous Diseases of the Extremities by Passive Congestion.**[3]—The procedure is very simple. It consists in placing a broad elastic band round the affected limb a few inches above the diseased part, firm enough to produce venous congestion. Bier recommends that this congestion should be kept up continuously, but others, notably A. G. Miller prefer to employ the pressure intermittently. The distal portion of the limb is supported by a common roller-bandage up to the affected part, so that the congested area is limited to the immediate neighbourhood of the disease. Bier records upwards of twenty cases which were treated at Professor Esmarch's wards at Kiel by this method, and reports that in most of the cases the improvement attained was very rapid and striking, and in no case did a change for the worse occur. Miller[4] has employed this remedy in tubercular joint and skin affections. The improvement in the latter was more marked than in the former, but a case of advanced strumous elbow joint disease, treated in the Edinburgh Royal Infirmary by this method, steadily improved. Miller suggests the employment of congestion treatment in the following cases: 1. Tubercular skin affections of the extremities. 2. Early tubercular disease of joints, combined with immobilisation or blisters, especially those in which time is of no great importance. 3. Instances of multiple tubercular disease in which radical treatment is inappropriate. The theory of action of this new remedy is based on Rokitansky's statement that "a congested lung possesses an immunity against tuberculosis." Mikulicz[5] reports very favourably of the plan. He gives a case—a white swelling of the knee previously treated without success by injections of iodoform—in which, on the application of Bier's treatment, the tubercular deposits disappeared in the course of eight weeks, the joint being freed from pain and active disease, but remaining much impaired in consequence of partial destruction and subsequent cicatrisation of the ligaments.

**After Treatment of Excision of the Elbow.**—Fleming[6] states that he does not employ any splint other than that formed by tightly-packed wood-wool dressing; that his first dressing is applied with the limb flexed, and that, as early as 48 hours after the operation, he begins passive movement. The apparatus by means of which he teaches patients to practice the movement of extension and flexion of the elbow consists of an upright board to which the patient's body is bandaged, and of an elastic band fixed to some object facing the patient and pulled upon by him. Pronation and supination are practised by rotating an instrument like a bar-bell held in the palm of the hand.

**Hip-Joint Disease and its Results.**—Bruns[7] sums up the results of the conservative treatment of 390 cases of tubercular coxitis. The results of the inquiry are as follows: 1. Many cases which had been looked upon as slight or incipient coxitis were found to have been only incurvation of the neck of the femur, an affection first described by Ernest Müller. 2. Cases of coxitis consequent upon infective osteo-myelitis of the femur are of far more frequent occurrence than has hitherto been supposed. 3. Tubercular coxitis affects almost exclusively persons below twenty years of age. 4. In one-third of the cases, tubercular coxitis ran its course without suppuration, in the other two-thirds abscesses and fistulæ are formed. 5. It is cured by the conservative treatment in 55 per cent. of cases, the duration of cure being four years. 6. A fatal end occurring in 40 per cent. of the cases is due to tubercular disease elsewhere, lardaceous disease, or septic infection. 7. In each individual case the prognosis depends on the presence or absence of suppuration. In non-suppurative forms the rate of recovery is 77 per cent., and the suppurative types 42 per cent. 8. Almost without exception in cured cases there is ankylosis with adduction or abduction of the limb.

In cases of rectangular ankylosis of the hip-joint a discussion of methods of remedying the deformity took place at the Clinical Society.[8] Mr. Heath showed two cases of rectangular ankylosis, in one of which division of the neck of the femur by Adams' method resulted in an excellently straight limb, the thigh having been previously fixed at a right angle. On a second patient, who had dislocation of the head of the femur, division of the bone between the trochanters (Sayre's operation) was performed with complete success. Keetley remarked that rectangular deformity could not be remedied except by removal of a wedge-shaped piece of bone with free division of the soft parts. Pickering Pick[9] in a clinical lecture on "Ankylosis of the Hip," discussed the various methods of remedying the deformity, and gave the preference to Adams' operation, but pointed out that there are certain cases in which this form of linear osteotomy is not applicable.

[1] Lancet, April 14th, 1894. [2] Brit. Med. Journ., May 5th, 1894. [3] Centralblatt. Für. Chir., 1892; Beilage, No. 32, p. 57. [4] Edin. Med. Jour., Feb. 1894. [5] Ep. B.M.J., April 21st, 1894. [6] Glasgow Med. Jour., May, 1894, p. 378. [7] Med. Week, April 27th, 1894, p. 193. [8] B. M. J., April 7th, 1894. [9] Clin. Jour., Feb. 7th, 1894, p. 231.

